# PCR-free whole exome sequencing: Cost-effective and efficient in detecting rare mutations

**DOI:** 10.1371/journal.pone.0222562

**Published:** 2019-09-13

**Authors:** Izumi Yamaguchi, Takashi Watanabe, Osamu Ohara, Yoshinori Hasegawa

**Affiliations:** Laboratory of Clinical Omics Research, Department of Applied Genomics, Kazusa DNA Research Institute, Chiba, Japan; Xiamen University, CHINA

## Abstract

In this study, we describe the development of a PCR-free whole exome sequencing method. Using this method, 2 μg DNA was sufficient for library preparation for whole exome sequencing. Furthermore, the method is simple and makes use of a commercial kit, with additional step of concentrating the captured library by ethanol precipitation. The accuracy of the PCR-free method was found to be equivalent to that of unique molecular identifier-corrected analysis method, which is the commonly used method to detect rare mutations. Thus, the PCR-free whole exome sequencing method is cost-effective as well as efficient in detecting rare mutations.

## Introduction

Whole exome sequencing (WES) with next-generation sequencing (NGS) is a powerful and cost-effective method for detecting mutations and small indels in all exons, and is widely utilized for analyses of inherited diseases [1–3]. The application of WES has been widened to analyses of somatic mutations [4–6]. However, polymerase chain reaction (PCR) error during library preparation is the most resistant obstacle for detection of de novo, low-frequency mutations [7–10]. Unique molecular identifier (UMI) has been developed to detect rare mutations with NGS [11]. UMI is a method that uses molecular tags to detect original sequence and quantify unique DNA and RNA molecules. Moreover, duplex sequencing, in which the tags present on each end of the paired reads are utilized, is a very powerful method with extremely low error rates [12–14]. Many kits with UMI are provided by manufacturers for DNA-Seq and RNA-Seq, and it has become easy for customers to utilize the kits, since most kits come with their own data analysis software. However, the use of these UMI-based kits becomes expensive, even those for WES. Furthermore, for clinical application, a large number of samples are required to check for rare de novo mutations in cancer tissues and quality inspection is required before transplantation of human iPS cells. Therefore, in the current study, we attempted to develop a PCR-free WES technique to detect rare mutations in a cost-effective manner.

## Materials and methods

### DNA sample

DNA sample of NA12878, which is a B-lymphocyte cell line established from peripheral blood mononuclear cells by transformation with Epstein-Barr virus, was purchased from Coriell institute.

### Ultrasonication of DNA

To use same condition of fragmented DNA by ultrasonication, a total of 20 μg DNA was taken in two sets of 10 μg DNA/tube and sheared using Covaris (Covaris, MA, USA) and used for every library preparation.

### Library preparation using PCR amplification

**Fig 1:**
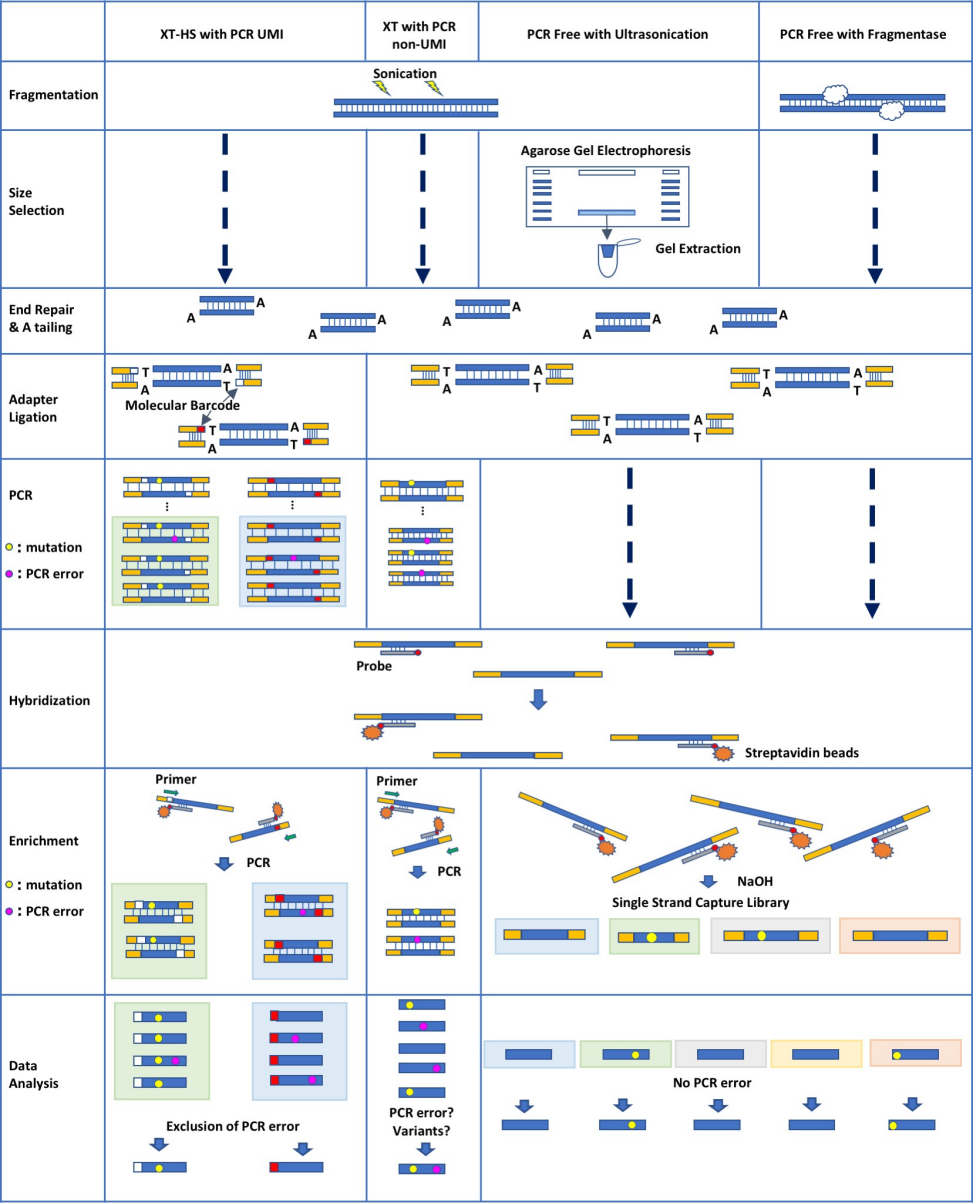
Workflow of four library preparation methods. Size selection process was skipped in XT-HS with PCR UMI, XT with PCR non-UMI, and PCR Free with Fragmentase library preparation methods. PCR process was not carried out in the PCR Free with Ultrasonication and PCR Free with Fragmentase methods.

Sonicated DNA (200 ng) was used for library preparation using SureSelect XT HS Reagents (HS-UMI) (Agilent, CA, USA) or SureSelect XT Reagents (XT-PCR) (Agilent) according to the manufacturer’s instructions (Fig 1 and Table 1).

**Table 1:** Conditions of four library preparation methods.

Library preparation method	XT-HS with PCR UMI	XT with PCR non-UMI	PCR Free with Ultrasonication	PCR Free with Fragmentase
(Abbreviation)	(HS-UMI)	(XT-PCR)	(PCRfree-Soni)	(PCRfree-Frag)
**Library preparation kit**	SureSelect XT-HS Kit	SureSelect XT Kit	KAPA HyperPrep Kit	NEBNext Ultra II FS DNA Library Prep Kit for Illumina
**UMI in the library**	Yes	No	No	No
**DNA polymerase**	Herculase	Herculase	-	-
**Fragmentation method**	Ultrasonication	Ultrasonication	Ultrasonication	Fragmentase
**DNA quantity used for library preparation (ng)**	200	200	4,000	2,000
**Cycle number of pre-capture PCR**	10	10	-	-
**DNA quantity used for hybridization (ng)**	750	750	3,000	1,000
**Cycle number of post-capture PCR**	10	10	-	-

### PCR-free library preparation of DNA sheared by Covaris and adaptor ligation using KAPA Hyper Prep Kit (PCRfree-Soni)

**Fig 2:**
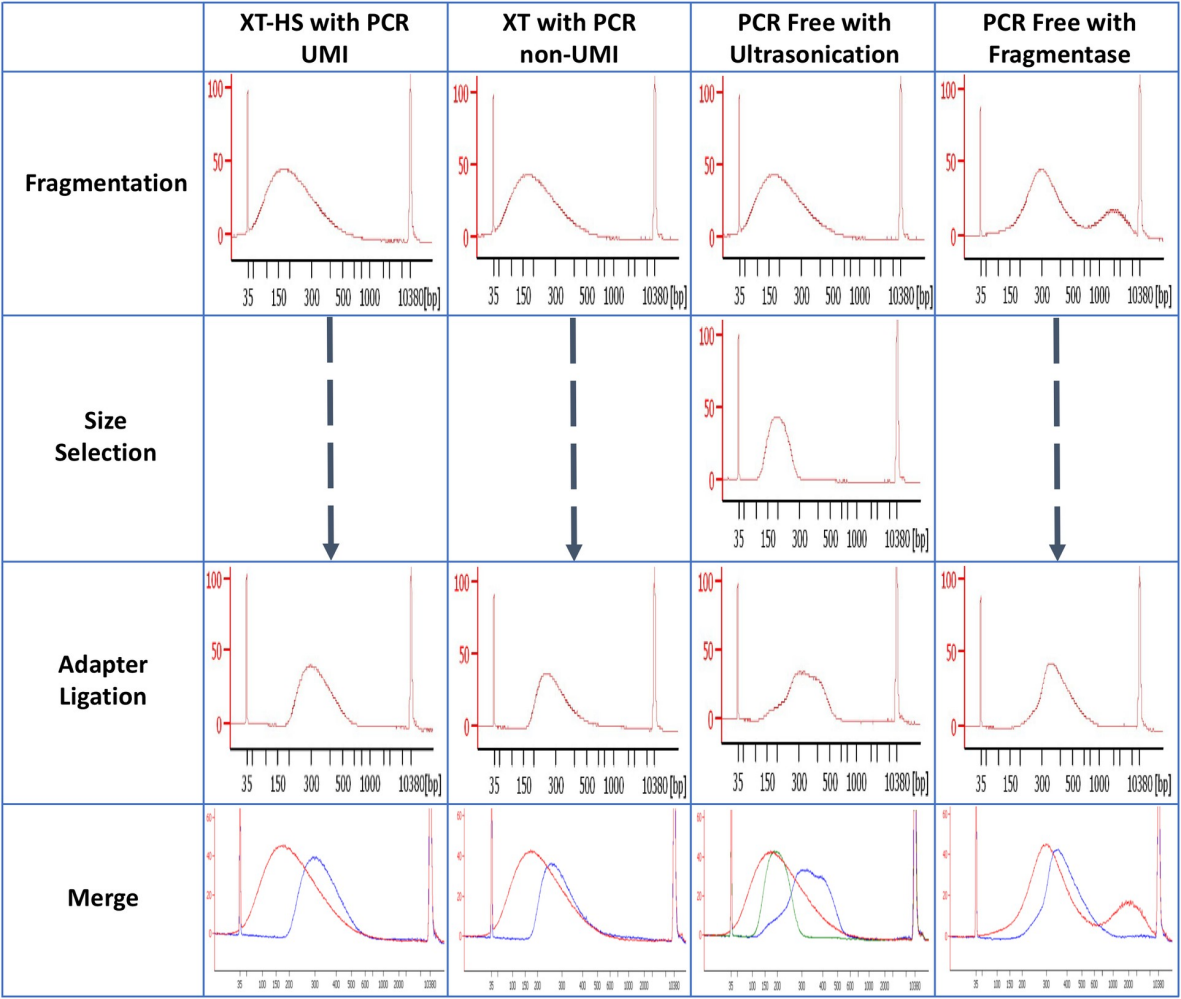
Libraries at each preparation process. All libraries were measured with Agilent BioAnalyzer 2100 using a High Sensitivity DNA chip. Size selection process was skipped in XT-HS with PCR UMI, XT with PCR non-UMI, and PCR Free with Fragmentase library preparation methods.

Approximately 20 μg sonicated DNA was size-selected using 2% agarose gel electrophoresis. The DNA from 100 bp to 300 bp was excised; the size-selected DNA was not stained with ethidium bromide (EtBr); instead, a precut marker DNA lane stained with EtBr was used as a guide for DNA size. Subsequently, the DNA was extracted from the gel using Wizard SV Gel and PCR Clean-Up System (Promega, WI, USA), according to the manufacturer’s instructions (Fig 1). The extracted DNA was then purified with AMpure XP (Beckman Coulter, USA) (Fig 2). Of the 4.43 μg of purified DNA, 4 μg DNA was subjected to end repair, A-tailing, and adaptor ligation with the KAPA Hyper Prep Kit (Kapa Biosystems, MA, USA), according to manufacturer’s instructions.

### PCR-free library preparation of DNA sheared enzymatically, followed by adaptor ligation using NEBNext Ultra II FS DNA Library Prep Kit (PCRfree-Frag)

Using the NEBNext Ultra II FS DNA Library Prep Kit for Illumina (NEB, MA, USA), 2 μg DNA was fragmented by DNA Fragmentase at 37°C for 15 min, followed by end repair, A-tailing, and adaptor ligation according to the manufacturer’s instructions, except that the ligation condition used was 4°C for 4 h to maximize the efficiency of adaptor ligation (Table 1).

### Target enrichment with SureSelect XT Human All Exon V5 kit

Input DNA amount for hybridization with the V5 kit was changed from 500 ng to 3000 ng for each library preparation method (Table 1). The quality and concentration of the libraries were verified using the Agilent 2100 Bioanalyzer and Qubit Fluorometer (Thermo Fisher Scientific, MA, USA), respectively. Target enrichment of all libraries was conducted according to the manufacturer’s instructions.

### Library quantification and sequencing of PCR-free captured libraries

Eluted PCR-free captured libraries (25 μL) were mixed with equal volume of 0.2 N NaOH and allowed to stand for 3 min at room temperature to separate the capture probes. After the released probes were removed with magnetic beads, the supernatant containing the single-stranded PCR-free libraries was neutralized with 50 μL of 200 mM Tris-HCl (pH 7.5). Next, 5 μg of glycogen (Thermo Fisher Scientific) was added to the collected PCR-free captured libraries as a co-precipitant. The libraries were precipitated by adding 100 μL of isopropyl alcohol and the obtained pellet was washed once with 70% ethanol and then dissolved in 35 μL or 15 μL RNase-free water for PCRfree-Soni and PCRfree-Frag, respectively. Library quantification was conducted by qPCR with GenNext NGS library quantification kit (TOYOBO, Japan). The libraries were directly mixed with another 10 pM UMI or non-UMI library diluted with HT1 buffer. All libraries were sequenced on an Illumina HiSeq 2500 system performing 100 bp paired-end reads. The raw data were deposited in the DNA Data Bank of Japan (DDBJ; accession nos. DRA008877, PRJDB8701).

### Exome sequence data analysis

All data analyses were conducted using the CLC genomics Workbench (CLCGW, v12, QIAGEN), except the UMI consensus reads, which were made with alignment reads sharing the same UMI, of HS-UMI using Strand NGS (v3.3, Agilent). Prior to importing into CLCGW, UMI reads of HS-UMI were attached to the head of read1 of HS-UMI, because the library prepared using SureSelect XT HS Reagent has a 10-bp UMI on the i5 index read. After importing into CLCGW and adaptor trimming from fastq reads, only the reads of HS-UMI were imported into Strand NGS. UMI consensus read sequences of HS-UMI were generated and those with family size (the number of reads in each family) less than 2 were removed. Then, the reads of HS-UMI were re-imported into CLCGW. All fastq reads were mapped to hg19 reference genome. Duplicate PCR reads were removed from the XT-PCR library. To analyze low-frequency mutations, basic variant detection operation was performed following local realignment operation. The results were corrected using VCF data of Illumina platinum genome NA12878 (https://www.illumina.com.cn/platinumgenomes.html) and compared among library preparation methods under the conditions of read coverage (the number of unique reads that include a given nucleotide) ≥ 20 and read count (the number of variant-supporting reads) ≥ 2.

## Results

### PCR-free WES

Firstly, to absolutely exclude fragmented DNA less than a sequence read length of 100 bp and easily confirm the status of adaptor ligation to the fragmented DNA, we began the experiment using DNA of 100 bp to 300 bp resulting from agarose-gel size selection for PCR-free library preparation. The size-selected DNA (4 μg) was ligated to the adaptor using KAPA Hyper prep kit. The adaptor ligation efficiency roughly estimated from the results of the bioanalyzer was about 70‒80% (Fig 2). We hybridized as much as 3000 ng library with the V5 probe. The captured library was denatured, followed by buffer exchange and concentration. Library quantification by qPCR showed that the estimated concentration of the libraries was 70.39 pM. Since this concentration was higher than the final concentration of the sequence library required for HiSeq (10 pM), we considered that these libraries could be sequenced by HiSeq. Therefore, we directly blended the PCR-free library with another 10 pM UMI or non-UMI library, and sequenced 76 million reads, with the sequence yield being about 70% of the yield estimated from the amount of input PCR-free library quantified by qPCR. On the other hand, the yield of UMI and non-UMI libraries sequenced with the PCR-free library was as expected by qPCR.

### Comparison among three library preparation methods

**Fig 3:**
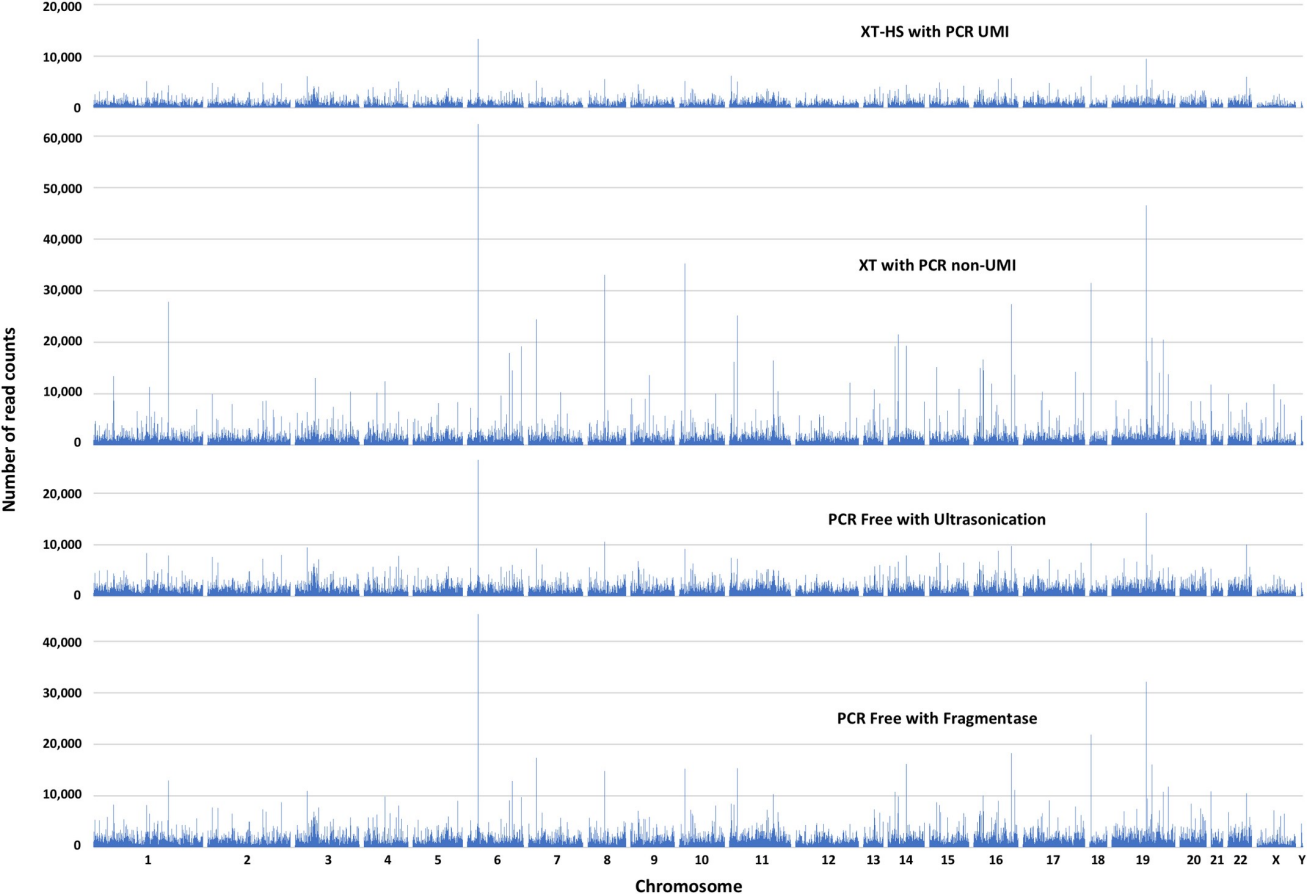
V5 target coverage maps of each library preparation method. Number of read counts at 230,418 target regions in the 50Mb of V5 exome kit.

**Fig 4:**
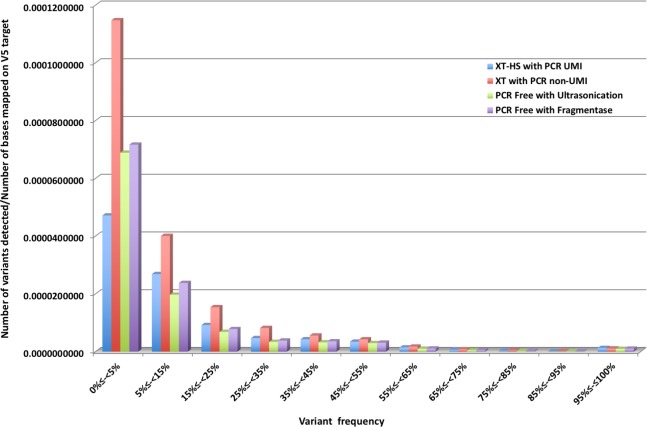
Frequency distribution of detected variants corrected with number of bases mapped on V5 target. Detected variants include single‐nucleotide variants (SNVs) and short insertions and deletions (indels).

To evaluate the accuracy of the PCR-free library method, we carried out three library preparation methods, PCRfree-Soni, HS-UMI, and XT-PCR, and compared the results of variant detection (Fig 1). For the HS-UMI method, we sequenced 359 million reads and 41.8 million consensus reads were obtained, of which only 102 million reads (28.4%) were used to make UMI consensus reads (Table 2). After adaptor trimming, mapping to hg19, removing duplicates (only XT-PCR), and making UMI consensus reads (only HS-UMI), the numbers of reads overlapping with the V5 target regions of HS-UMI, XT-PCR, and PCRfree-Soni were 36,808,046, 63,572,153, and 63,936,438 respectively (Table 2). After local realignment operation, basic variant detection operation was conducted. Reads were mapped throughout the V5 target regions of all three library preparation methods (Fig 3). The coverage map of XT-PCR showed larger variation than that of PCRfree-Soni although the number of mapped reads of these two was approximately equal. The corrected frequency of detected SNP and small indel of PCRfree-Soni was almost the same as that of HS-UMI (Table 2 and Fig 4) and was lower than that of XT-PCR. These results showed that the accuracy of PCR-free method was superior to that of normal exome sequencing with PCR (XT-PCR) and equal to that of UMI corrected method (HS-UMI).

**Table 2:** Statistics of sequence reads.

Library preparation method	XT-HS with PCR UMI	XT with PCR non-UMI	PCR Free with Ultrasonication	PCR Free with Fragmentase
(Abbreviation)	(HS-UMI)	(XT-PCR)	(PCRfree-Soni)	(PCRfree-Frag)
**Total Reads**	358,986,380	84,000,008	76,060,154	79,722,282
**UMI consensus reads (family size ≥ 2)**	41,781,418	-	-	-
**Mapped reads on V5 target**	36,808,046	70,242,571	63,936,438	67,993,050
**Duplicate reads removed**	-	63,572,153	-	-
**Number of bases mapped on V5 target**	3,370,645,303	4,710,769,927	5,193,862,316	4,787,140,030
**Average coverage across all V5 target regions**	65.8	92.1	101.8	93.7
**Minimum coverage of target regions 10×**	89.18%	97.73%	94.88%	94.22%
**Minimum coverage of target regions 20×**	80.10%	94.18%	89.76%	88.14%
**Minimum coverage of target regions 40×**	60.43%	80.44%	78.99%	74.84%
**Minimum coverage of target regions 100×**	20.01%	34.67%	41.50%	35.85%
**Number of variants detected**	334,216	905,983	559,011	557,950
**Number of variants detected/Number of bases mapped on V5 target**	0.992 × 10^−4^	1.923 × 10^−4^	1.076 × 10^−4^	1.166 × 10^−4^

### PCR-free library preparation using DNA sheared by Fragmentase

We confirmed that PCR-free WES is viable as stated above. Next, we tried to use DNA Fragmentase for PCR-free library preparation, because commercial DNA Fragmentase-based kits, such as KAPA Hyper plus kit and NEBNext Ultra II FS DNA Library Prep Kit for Illumina, showed higher adaptor-ligated library yield than did the covaris-sheared DNA processed kits. Starter DNA amount was reduced to 2 μg, and the shearing condition was adapted to lengthen DNA insert (Fig 2). The estimated concentration of 15 μL of the final library showed 137.11 pM. The total yield of the final library was enough to sequence over 200 million reads by HiSeq. We then sequenced 79.7 million reads, which again showed about 70% of the estimated yield by qPCR. The proportion of reads overlapping with the V5 target regions between PCRfree-Frag and PCRfree-Soni was almost the same, and the accuracy of the two methods was also similar (Table 2). These results showed that the performance of PCRfree-Frag was almost equal to that of PCRfree-Soni.

## Discussion

Our results showed that 2 μg DNA is sufficient to conduct PCR-free WES analysis, with the rate of mutation detection equaling that achieved with UMI-based methods. The PCR-free WES method described here satisfied the practical level required for detection of cancer specific mutations and iPS cell quality check. PCR-free method was shown to be effective not only in detection of rare mutation but also in detection of long repeat expansions [15]. We could conduct PCR-free WES analysis with less amount of DNA (500 ng– 1000 ng) in combination with longer read length, such as 125 bp, 150 bp, and 250 bp by HiSeq.

For practical analysis, it is desirable to utilize the consensus reads of UMI family size more than 2 [16]. The members of UMI libraries amplified by PCR from 200 ng DNA were too large to make UMI consensus reads efficiently, and the reads generated from 359 million fastq reads were very few (6,045,390 reads). Of course, if we use 10 ng DNA for HS-UMI, more UMI consensus reads would be possible. However, our goal was to establish a cost-effective detection method of rare somatic mutation using WES; therefore, reducing DNA amount is not appropriate for the purpose of detecting rare mutations.

Notably, the sequence yield of PCR-free captured libraries showed reproducibility of about 70% of that estimated by qPCR quantification. This might be due to the fact that the DNA standard in the qPCR kit was double-stranded DNA. Nonetheless, we believe that the PCR-free WES method is powerful and cost-effective for screening a large number of samples to detect rare mutation and small indels in cancer tissues and human iPS cells.
